# Comparative and Phylogenetic Analysis Based on the Chloroplast Genome of *Coleanthus subtilis* (Tratt.) Seidel, a Protected Rare Species of Monotypic Genus

**DOI:** 10.3389/fpls.2022.828467

**Published:** 2022-02-24

**Authors:** Jing Ren, Jing Tian, Hui Jiang, Xin-Xin Zhu, Fredrick Munyao Mutie, Vincent Okelo Wanga, Shi-Xiong Ding, Jia-Xin Yang, Xiang Dong, Ling-Ling Chen, Xiu-Zhen Cai, Guang-Wan Hu

**Affiliations:** ^1^College of Life Sciences, Hunan Normal University, Changsha, China; ^2^CAS Key Laboratory of Plant Germplasm Enhancement and Specialty Agriculture, Wuhan Botanical Garden, Chinese Academy of Sciences, Wuhan, China; ^3^Sino-Africa Joint Research Center, Chinese Academy of Sciences, Wuhan, China; ^4^University of Chinese Academy of Sciences, Beijing, China; ^5^College of Life Sciences, Xinyang Normal University, Xinyang, China

**Keywords:** *Coleanthus subtilis*, chloroplast genome, comparative analysis, phylogeny, monotypic genus

## Abstract

*Coleanthus subtilis* (Tratt.) Seidel (Poaceae) is an ephemeral grass from the monotypic genus *Coleanthus* Seidl, which grows on wet muddy areas such as fishponds or reservoirs. As a rare species with strict habitat requirements, it is protected at international and national levels. In this study, we sequenced its whole chloroplast genome for the first time using the next-generation sequencing (NGS) technology on the Illumina platform, and performed a comparative and phylogenetic analysis with the related species in Poaceae. The complete chloroplast genome of *C*. *subtilis* is 135,915 bp in length, with a quadripartite structure having two 21,529 bp inverted repeat regions (IRs) dividing the entire circular genome into a large single copy region (LSC) of 80,100 bp and a small single copy region (SSC) of 12,757 bp. The overall GC content is 38.3%, while the GC contents in LSC, SSC, and IR regions are 36.3%, 32.4%, and 43.9%, respectively. A total of 129 genes were annotated in the chloroplast genome, including 83 protein-coding genes, 38 tRNA genes, and 8 rRNA genes. The *accD* gene and the introns of both *clpP* and *rpoC1* genes were missing. In addition, the *ycf1*, *ycf2*, *ycf15*, and *ycf68* were pseudogenes. Although the chloroplast genome structure of *C*. *subtilis* was found to be conserved and stable in general, 26 SSRs and 13 highly variable loci were detected, these regions have the potential to be developed as important molecular markers for the subfamily Pooideae. Phylogenetic analysis with species in Poaceae indicated that *Coleanthus* and *Phippsia* were sister groups, and provided new insights into the relationship between *Coleanthus*, *Zingeria*, and *Colpodium*. This study presents the initial chloroplast genome report of *C. subtilis*, which provides an essential data reference for further research on its origin.

## Introduction

*Coleanthus subtilis* (Tratt.) Seidel is a rare grass in the monotypic genus *Coleanthus* Seidl, which can be recognized by its rosette-like arrangement of stems, the wide leaf sheaths and curved leaves (Richert et al., 2016). It has a wide but disjunctive distribution area and has been recorded in west-central Europe, southern Norway, Russia, China, United States, and Canada (Richert et al., 2014). It occurs mainly on wet and muddy habitats, growing along streams or rivers (Taran, 1994). Its secondary habitats are artificial ponds and reservoirs, where changes in water level expose bare and moist surfaces that give the seeds the opportunity to germinate (Hejný, 1969; Woike, 1969; Richert et al., 2014). It is an ephemeral grass whose life cycle lasts only a few weeks and requires high levels of moisture and nutrients from germination to reproduction. Moreover, in order to germinate, a diurnal temperature difference of at least 20°C is necessary (Hejný, 1969; Richert et al., 2014). Destruction of favorable habitats in regions such as Europe have threatened the survival of *C. subtilis*. With the development of fisheries and tourism, ponds and reservoirs are becoming increasingly populated with anglers, which affects the secondary habitat of *C. subtilis*. Furthermore, the frequency and timing of ponds and reservoirs drainage also influence the reproductive cycle of *C. subtilis*, as prolonged periods without drainage may limit seed germination and result in failure to renew the seed bank (Richert et al., 2014). The strict conditions for reproduction combined with habitat destruction have led to a sharp decline in the populations of *C. subtilis*, hence it is protected at both national and international levels. For example, it is listed in Annexes II and IV of the Habitats Directive by the European Union Organization and is also documented in Appendix I of the Berne Convention (John et al., 2010). Besides, *C. subtilis* is considered a species in need of conservation in other countries, such as the Czechia (Grulich, 2012) and North America (Catling, 2009). In China, it also has been listed as a second-class national key protected wild plant^1^.

*Coleanthus subtilis* has long been of interest to researchers due to its special distribution pattern, strict habitat requirements and unique inflorescence structure (Kurchenko, 2006). *C. subtilis* has a remarkable ability to reappear in its previous habitats after long time intervals, which may be related to the hypothesis that its seeds can remain viable in the soil for decades (Hejný, 1969; Richert et al., 2014). For example, it was rediscovered in 2001 at Volkhov Shoal, where it was mistakenly thought to have been extinct for 70 years (Yurova, 2001). In 2021, we found it in Harbin after an interval of nearly 100 years. In addition, *C. subtilis* was collected on the banks of the Yangtze River in Wuhan, where its distribution has never been recorded before. The factors responsible for this particular distribution pattern are unclear.

Based on morphological studies, *C. subtilis* was once considered a member of the tribe Agrostideae because of its distinctive inflorescence, which has flowers aggregated in bunches and with staminodes (Gnutikov et al., 2020). In addition, it has been placed near the genera *Alopecurus* and *Mibora*, although it does not share common features with these two (Gnutikov et al., 2020). However, some researchers believe that there is a close relationship between the genus *Coleanthus* and the genus *Phippsia* because of the similarities in morphology and ecological preferences (Tzvelev, 1976; Gnutikov et al., 2020). Soreng et al. (2003) proposed a new subfamily called Puccinelliinae based on molecular phylogenetic analysis and more thorough morphological examination of Poaceae, which are characterized by thin membranous lemmas with hyaline apex and glabrous margins. Hoffmann et al. (2013) placed *C. subtilis* in the Puccinelliinae using DNA sequence data of the ribosomal internal transcribed spacer (ITS). Subsequently, the subtribe Puccinelliinae was renamed as Coleanthinae after the addition of *Coleanthus* (Soreng et al., 2015). The use of chloroplast genes or fragments (*matK*, *ndhF*, and *trnL-trnF*) to explore the phylogenetic position of *C. subtilis* showed that it is most closely related to the genus *Phippsia* and that both are sister groups to other genera in the subtribe Coleanthinae of the subfamily Pooideae (Gnutikov et al., 2020), but opinions differ on the composition of this subtribe (Soreng et al., 2003, 2015; Gnutikov et al., 2020; Tkach et al., 2020). The whole chloroplast genomes provide more complete genetic information than single gene fragments to enable better discovery of interspecific genetic resources and understanding of evolutionary history (Wariss et al., 2018). However, to date, no studies have explored the phylogenetic position of *C. subtilis* with the help of complete chloroplast genomes, which affects our comprehensive understanding of its phylogeny.

Compared with nuclear and mitochondrial genome, chloroplast genomes are characterized by moderate nucleotide substitution rates, structural simplicity and uniparental inheritance (Burke et al., 2012; Ruhfel et al., 2014; Yang et al., 2019), which makes them ideal resources in phylogenetic studies at different levels and a common tool for species identification (Chen et al., 2018; Yu et al., 2021). Its structure is relatively stable and contains a large amount of genetic information, which is considered a valuable data resource for solving complex evolutionary relationships (Parks et al., 2009; Moore et al., 2010; Oldenburg and Bendich, 2016). At the same time, it has a promising future in molecular marker studies, as some genes are often used in DNA barcoding for species identification, such as *rbcL* and *matK* (Hollingsworth, 2011). In addition, chloroplast genomes have been widely used in plant genetic diversity and conservation studies, since they can provide more complete genetic information compared to individual gene fragments, therefore facilitating better resolution of evolutionary relationships among species (Wariss et al., 2018). Next-generation sequencing (NGS) technology provides an efficient and cost-effective method for chloroplast genome assembly, which greatly enriches chloroplast genome information and provides sufficient data for plant phylogenetic studies (Cronn et al., 2008; Tangphatsornruang et al., 2010). Despite this, the chloroplast genome of *C. subtilis* has not been reported to date, which limits its development of genetic information discovery and phylogenetic studies.

Therefore, the purpose of this study is to (a) provide the first report on the chloroplast genome of the genus *Coleanthus* and conduct a comparative genomic analysis with other species in the subfamily Pooideae; (b) make the first attempt to reconstruct the phylogeny of the subfamily Pooideae based on chloroplast genome information to explore the phylogenetic position of *C. subtilis*; (c) identify highly variable loci to provide useful information for future development of molecular markers in *C. subtilis*.

## Materials and Methods

### Sampling, Extraction, and Genome Sequencing

The materials of *Coleanthus subtilis* were collected from Harbin, China, in June 2021, and subsequently deposited in the Herbarium of the Wuhan Botanical Garden (HIB), Chinese Academy of Sciences (China), with herbarium number ZXX21129. For drying and long-term preservation of molecular samples, fresh leaves were preserved in silica gel (Chase and Hills, 2019). The complete genomic DNA of *C. subtilis* chloroplast was extracted using a modified CTAB procedure (Allen et al., 2006) and then sequenced at Novogene Co., Ltd. (Beijing, China) with Illumina paired-end technology platform. Purified high-quality genomic DNA was broken into short fragments of approximately 350 bp, and paired-end (PE) libraries were constructed by adding A-tails, PCR amplification and other steps, followed by sequencing in 150 bp paired-end mode on an Illumina HiSeq 2500 platform. The final number of raw reads obtained was 36,062,743 and that of clean reads after filtering was 35,335,540. The raw data has been uploaded to the NCBI database (BioProject ID: PRJNA802068).

### Assembly and Annotation of Chloroplast Genome

Get Organelle v1.7.4 (Jin et al., 2020) was used to assemble the chloroplast genome with default parameters. The low-quality reads and adapters were first filtered, then a *de novo* assembly performed, and the results were further purified to generate the complete chloroplast genomes. The results were visualized with Bandage (Wick et al., 2015). The Plastid Genome Annotator (PGA) software (Qu et al., 2019) was used to perform the annotation of the entire chloroplast genome, and in addition to using *Amborella trichopoda* as the reference genome, some Poaceae species were also selected to enhance the credibility of the annotation results. Furthermore, to ensure the accuracy of the annotation results, the genome was also annotated simultaneously with the help of GeSeq online tool^2^ (Tillich et al., 2017).

The check of annotated genes was implemented in the software Geneious-v10.2.3 (Kearse et al., 2012), which was used to further verify and refine the annotation results and to manually correct errors detected in gene annotation. Special attention was paid to some genes located at the boundaries and the highly variable genes, such as *ndhF*, *ndhK*, *ycf2*, *accD*, etc. The circular chloroplast genome map of *Coleanthus subtilis* was drawn and visualized using OGDraw online tool^3^ (Greiner et al., 2019). Lastly, the annotated sequence was submitted to GenBank on the NCBI website, with an accession number OL692806.

### Comparative Analysis of the Chloroplast Genome

The chloroplast genome characteristics of *Coleanthus subtilis* were analyzed in Geneious-v10.2.3 software by comparing chloroplast genomes with those of Poaceae species downloaded from the NCBI database (Supplementary Table 1). A total of 24 species representing 10 subtribes (5 tribes) were used for the comparative analysis of chloroplast genomes. Additionally, to determine genomic divergence among these species, genomic similarity analysis was performed using the Glocal alignment program (shuffle-LAGAN mode) in mVISTA (Brudno et al., 2003; Frazer et al., 2004) with *C. subtilis* as the reference. The SC/IR boundary analysis was done using the IRscope (Amiryousefi et al., 2018) to observe the contraction and/or the expansion of the genes at the borders. For the codon usage bias analysis, MEGA 7.0 software (Kumar et al., 2016) was chosen to calculate relative synonymous codon usage (RSCU) values based on the coding sequences (CDS regions).

### Analysis of Repeats and Nucleotide Diversity

The REPuter tool^4^ (Kurtz et al., 2001) was used to identify repeats including forward, reverse, palindrome, and complement sequences. When the Hamming distance is equal to 3, the length and identity of repeats are limited to ≥30 bp and >90%, respectively. The simple sequence repeats (SSRs) were analyzed using the MISA (Beier et al., 2017) with the basic repeat setting: a threshold of 10, 5, 4, 3, 3, and 3 for mono-, di-, tri-, tetra-, penta-, and hexa-nucleotides, respectively. The DnaSP-v5.10 software (Librado and Rozas, 2009) was used to calculate nucleotide variability (*Pi*) values and variable sites using the aligned chloroplast genome sequences with a window length of 600 bp and a step size of 200 bp.

### Substitution Rate Analysis

The EasyCodeML program in PAML package (Gao et al., 2019) was utilized to identify positive sites in protein-coding genes to quantify selection pressure. This software provided four site models (M0 vs. M3, M1a vs. M2a, M7 vs. M8, and M8a vs. M8), Bayes Empirical Bayes (BEB) analysis (Yang et al., 2005) and Naive Empirical Bayes (NEB) analysis were performed in each model to measure the loci with positive selection pressure.

### Phylogenetic Analysis

To understand the phylogenetic position of *Coleanthus subtilis* in the family Poaceae and its affinities with other species, a phylogenetic tree was reconstructed using the Maximum Likelihood (Felsenstein, 1981) and Bayesian Inference analysis (Huelsenbeck et al., 2001). This was based on 76 shared protein-coding genes of the Chloroplast genome from a total of 53 species from 26 genera in Poaceae, with *Acidosasa purpurea* as the outgroup (Supplementary Table 1). Each protein-coding sequence was first aligned in the software MAFFT-v7.409 (Katoh and Standley, 2013), followed by removing the stop codon and discarding the bad fragment with the Gblock program (Talavera and Castresana, 2007) and later concatenated using the concatenated in-built PhyloSuite program (Zhang et al., 2020). ML analysis in IQ-tree and BI analysis in MrBayes were used to infer phylogenetic relationships. The best-fit models for each of the two analyses were found in Model Finder (Kalyaanamoorthy et al., 2017) according to the Bayesian Information Criterion (BIC), and the most suitable model for Bayesian analysis was detected as GTR + F + I + G4, while GTR + F + R3 was used for the Maximum Likelihood analysis. Subsequently, the BI tree was constructed by the software MrBayes-3.2.6 (Ronquist et al., 2012) for 1,000,000 generations, sampling every 1000 generations, and the software IQ–TREE was implemented to construct the ML tree with bootstrap replications of 1000 (Lam-Tung et al., 2015). The phylogenetic trees were visualized in the software Figtree-v1.4.4^5^. Both phylogenetic trees were combined manually using AI software based on consistent topological structures. The results were imported into the software Figtree-v1.4.4 to view the generated phylogenetic trees and to enhance their visualization. Considering the consistent topology, the phylogenetic trees constructed by both methods were manually combined in the AI software.

## Results

### Chloroplast Genome Features

**FIGURE 1:**
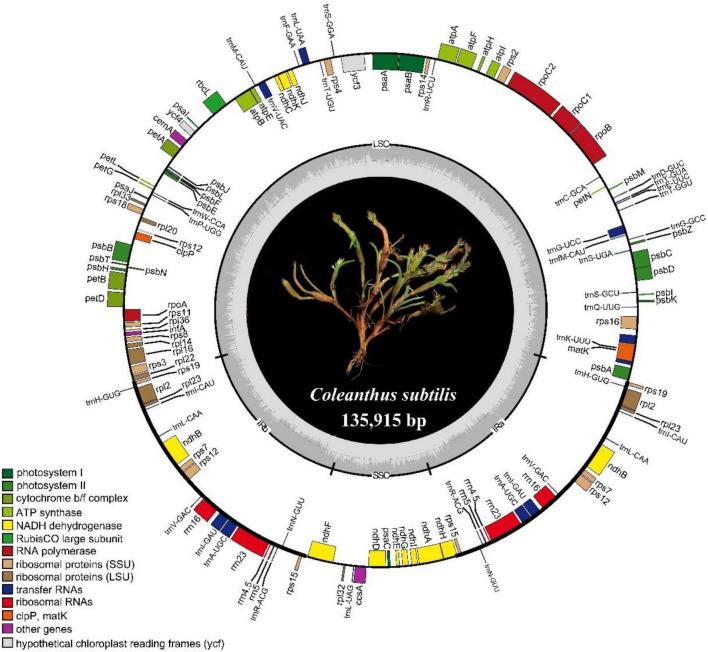
Chloroplast genome map of *C. subtilis*. The genes located inside the circles are transcribed in a clockwise direction, while those outside the circle are transcribed counterclockwise. Different colored genes represent different functions, as shown in the legend at the bottom left. The inverted boundaries and GC content are drawn in the inner circle.

The chloroplast genome of *Coleanthus subtilis* is 135,915 bp in size and consists of four regions that together form a loop structure. These four regions are the large single copy region (LSC) of 80,100 bp, a small single copy region (SSC) of 12,757 bp, and two inverted repeat regions (IR) of 21,529 bp in length, respectively. In addition, a pair of inverted repeat regions separate the two single-copy regions (Figure 1 and Table 1). GC content varies in different regions of the chloroplast genome. The highest GC content of 43.9% was found in the IR regions of *C. subtilis*, while the two single copy regions had 36.3% (LSC) and 32.4% (SSC) (Table 1).

**TABLE 1:** Features of the chloroplast genomes of *C. subtilis* and other Poaceae species.

Species	Genome length (bp)				GC content (%)				Gene number			
	Total	LSC	SSC	IR	Total	LSC	SSC	IR	Total	PCG	tRNA	rRNA
*Coleanthus subtilis*	135915	80100	12757	21529	38.3	36.3	32.4	43.9	129	83	38	8(4)
*Phippsia algida*	135613	79805	12750	21529	38.3	36.3	32.4	43.9	129	83	38	8(4)
*Puccinellia nuttalliana*	135353	79594	12727	21516	38.3	36.3	32.6	43.9	129	83	38	8(4)
*Sclerochloa dura*	135086	79683	12609	21397	38.3	36.2	32.6	43.9	129	83	38	8(4)
*Zingeria biebersteiniana*	135943	80121	12390	21491	38.3	36.2	32.7	43.9	129	83	38	8(4)
*Agrostis gigantea*	136705	80604	12769	21666	38.5	36.4	32.8	44.0	129	83	38	8(4)
*Alopecurus japonicus*	136408	80511	12835	21531	38.3	36.2	32.4	43.9	129	83	38	8(4)
*Ammophila breviligulata*	136726	80711	12701	21657	38.6	36.5	32.9	44.1	129	83	38	8(4)
*Anthoxanthum odoratum*	135551	79626	12671	21627	38.2	36.1	32.5	43.9	129	83	38	8(4)
*Avena barbata*	135946	80111	12625	21605	38.5	36.4	32.6	44.0	129	83	38	8(4)
*Brachypodium stacei*	136330	81252	12666	21206	38.6	36.6	32.7	44.1	129	83	38	8(4)
*Briza maxima*	136823	79707	12722	22917	38.3	36.2	32.6	43.7	129	83	38	8(4)
*Bromus vulgaris*	136934	80964	12566	21702	38.3	36.3	32.3	43.9	128	83	37	8(4)
*Calamagrostis pickeringii*	136682	80660	12688	21667	38.6	36.5	32.9	44.0	129	83	38	8(4)
*Castellia tuberculosa*	133798	78819	12497	21241	38.4	36.3	32.6	43.9	129	83	38	8(4)
*Colpodium humile*	133608	78636	12474	21249	38.3	36.2	32.7	43.9	129	83	38	8(4)
*Festuca altissima*	135272	79826	12598	21424	38.4	36.4	32.8	43.9	129	83	38	8(4)
*Hierochloe odorata*	136395	80645	12466	21642	38.5	36.4	33.0	44.0	129	83	38	8(4)
*Lolium multiflorum*	135175	79848	12485	21421	38.3	36.1	32.4	43.9	129	83	38	8(4)
*Melica mutica*	134710	80478	12570	20831	38.5	36.5	32.8	44.0	129	83	38	8(4)
*Phalaris coerulescens*	135794	79728	12760	21653	38.5	36.4	32.9	44.0	129	83	38	8(4)
*Phleum alpinum*	135568	80009	12823	21368	38.4	36.3	32.6	44.0	129	83	38	8(4)
*Poa diaphora*	135466	79629	12685	21576	38.3	36.2	32.4	43.8	129	83	38	8(4)
*Stipa purpurea*	137370	81202	12842	21663	38.8	36.9	32.9	44.0	129	83	38	8(4)

A total of 129 genes were annotated in the chloroplast genome of *C. subtilis*, with 83 protein-coding genes (PCGs), 38 tRNA genes, and 8 rRNA genes. In addition, the *accD* gene was found missing in the chloroplast genome, while *ycf1*, *ycf2*, *ycf15*, and *ycf68* were pseudogenes (Table 2). These genes were divided into three groups based on their different functions. Nineteen genes were observed to replicate in the inverted repeat regions, seven of which were PCGs (*ndhB*, *rpl2*, *rpl23*, *rps7*, *rps12*, *rps15*, *rps19*), eight were tRNA genes (*trnA-UGC*, *trnI-CAU*, *trnI-GAU*, *trnH-GUG*, *trnL-CAA*, *trnN-GUU*, *trnR-ACG*, and *trnV-GAC*) and the remaining four genes were rRNA (*rrn4.5*, *rrn5*, *rrn16*, and *rrn23*). In addition, the largest number of genes in the LSC region was 82, while only 11 genes were located in the SSC region. More interestingly, all rRNA genes were distributed in the IR regions (Supplementary Table 2). We identified 15 genes containing one intron in *C. subtilis*, with six being tRNAs and nine being PCGs. It’s important to highlight that *trnK-UUU* had the longest intron with 2480 bp, which completely wrapped the *MatK* gene. Meanwhile, the *ycf3* gene contained two codons with lengths of 774 bp and 726 bp (Supplementary Table 3).

**TABLE 2:** List of the annotated genes in the chloroplast genomes of *C. subtilis*.

Category	Groups of genes	Name of genes
Self-replication	Ribosomal RNA	*rrn4.5* ^c^, *rrn5* ^c^, *rrn16* ^c^, *rrn23* ^c^
	Transfer RNA	*trnA-UGC* ^a,c^, *trnC-GCA*, *trnD-GUC*, *trnE-UUC*, *trnF-GAA*, *trnfM-CAU*, *trnG-GCC*, *trnG-UCC*^a^, *trnH-GUG* ^c^, *trnI-CAU*^c^, *trnI-GAU* ^a,c^, *trnK-UUU* ^a^, *trnL-CAA* ^c^, *trnL-UAA* ^a^, *trnL-UAG*, *trnM-CAU*, *trnN-GUU* ^c^, *trnP-UGG*, *trnQ-UUG*, *trnR-UCU*, *trnR-ACG* ^c^, *trnS-UGA*, *trnS-GCU*, *trnS-GGA*, *trnT-GGU*, *trnT-UGU*, *trnV-UAC* ^a^, *trnV-GAC*^c^, *trnW-CCA*, *trnY-GUA*
	Small subunit of ribosome	*rps2*, *rps3*, *rps4*, *rps7* ^c^, *rps8*, *rps11*, *rps12* ^a,c^, *rps14*, *rps15* ^c^, *rps16* ^a^, *rps18*, *rps19* ^c^
	Large subunit of ribosome	*rpl2* ^a,c^, *rpl14*, *rpl16* ^a^, *rpl20*, *rpl22*, *rpl23*^c^, *rpl32*, *rpl33*, *rpl36*
	RNA polymerase subunits	*rpoA*, *rpoB*, *rpoC1*, *rpoC2*
Photosynthesis	Photosystem I	*psaA*, *psaB*, *psaC*, *psaI*, *psaJ*, *ycf3* ^b^, *ycf4*
	Photosystem II	*psbA*, *psbB*, *psbC*, *psbD*, *psbE*, *psbF*, *psbH*, *psbI*, *psbJ*, *psbK*, *psbL*, *psbM*, *psbN*, *psbT*, *psbZ*
	Subunits of cytochrome	*petA*, *petB* ^a^, *petD*^a^, *petG*, *petL*, *petN*
	ATP synthase	*atpA*, *atpB*, *atpE*, *atpF* ^a^, *atpH*, *atpI*
	NADH-dehydrogenase	*ndhA* ^a^, *ndhB* ^a,c^, *ndhC*, *ndhD*, *ndhE*, *ndhF*, *ndhG*, *ndhH*, *ndhI*, *ndhJ*, *ndhK*
Other genes	Rubisco large subunit	*rbcL*
	Translational initiation factor	*infA*
	Maturase K	*matK*
	Envelope membrane protein	*cemA*
	Proteolysis	*clpP*
	Cytochrome c biogenesis	*ccsA*

*^a^Genes with one intron. ^b^Genes with two introns. ^c^Two gene copied in IR regions.*

**FIGURE 2:**
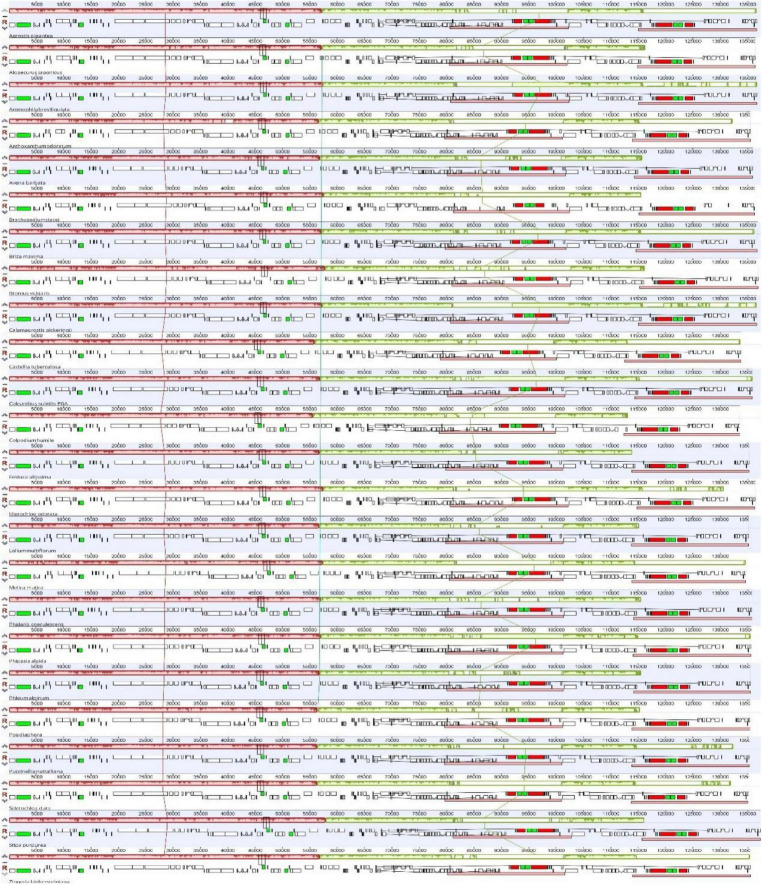
Comparison of the chloroplast genome structures among 24 Poaceae species. The different colored squares represent different types of genes. Black represents transfer RNA (tRNA), or green if the tRNA has introns (rRNA). Red represents ribosomal RNA, while white represents protein coding genes (PCGs).

The chloroplast genome of *C. subtilis* showed high similarities with other Poaceae species in terms of genome length and structure, GC content and gene number. The complete genomes length varied from 133608 bp (*Colpodium humile*) to 137370 bp (*Stipa purpurea*), LSC from 78636 bp (*Colpodium humile*) to 81252 bp (*Brachypodium stacei*), SSC from 12390 bp (*Zingeria biebersteiniana*) to 12842 bp (*Stipa purpurea*), and IR from 20831 bp (*Melica mutica*) to 22917 bp (*Briza maxima*) (Table 1). The overall GC content was around 38.5%, and each of the four regions also differed only insignificantly. In particular, gene number and composition were almost identical in 24 species, with only the *trnL-UAA* gene missing in *Bromus vulgaris*. In addition, no structural rearrangements were found in any of them (Figure 2).

### Junction Characteristics

**FIGURE 3:**
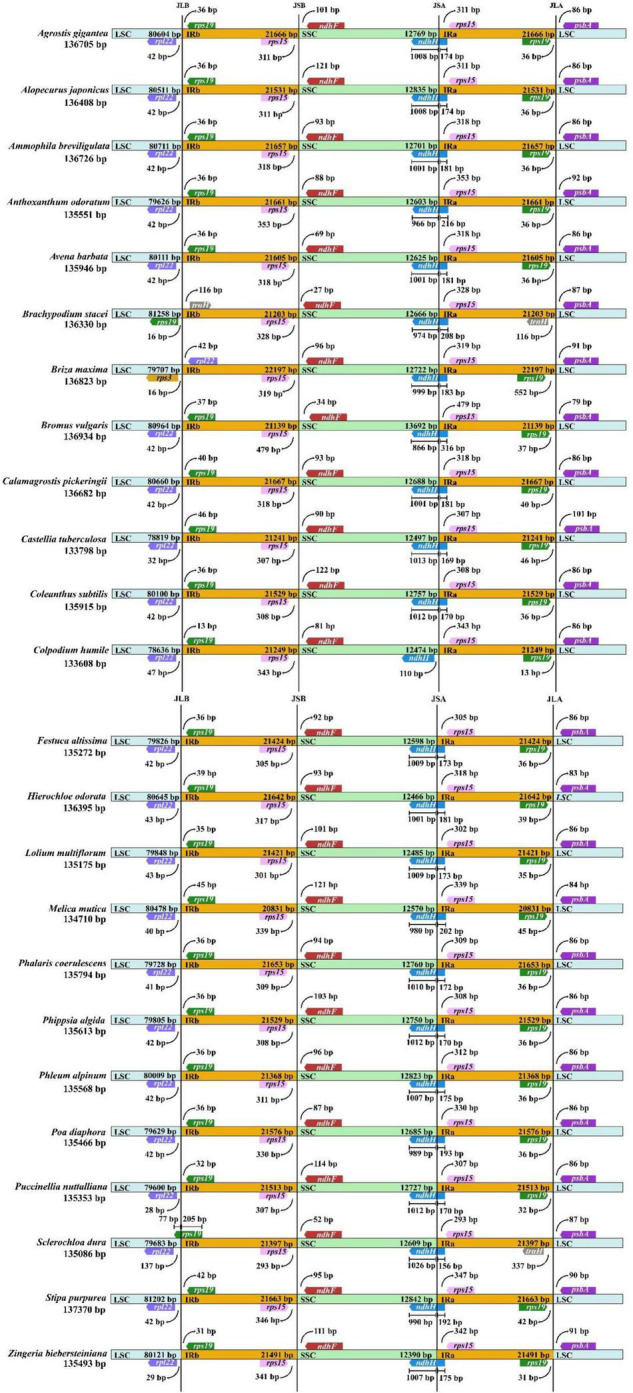
A plot of comparative analysis of the boundary features of the 24 Poaceae species. The comparative regions are the boundaries of large single copy (LSC), small single copy (SSC) and inverted repeat (IR) regions.

To observe the variation of IR boundaries, we did a comparative analysis of the junction structure based on the chloroplast genomes of *Coleanthus subtilis* and 23 other Poaceae species (Figure 3). The results showed that their boundary features were similar, the genes found at the nodes were mainly *rpl22*, *rps19*, *rps15*, *ndhF*, *ndhH*, and *psbA*. The *rps19* and *rps15* genes were replicated and fully embedded in the IR region, with lengths of 13–46 bp and 293–479 bp from the two IR/LSC boundaries, respectively. The *ndhF* genes were located entirely on the left of the IRb/SSC and were 27 to 122 bp from this boundary. Also, the *ndhH* gene occupied the IRa/SSC junction and was overwhelmingly located within the SSC region, with only a small portion of 156 to 316 bp extending into the IRa region. It should be noted that the *ndhH* gene of *Colpodium humile* was slightly shorter in length and was therefore completely encapsulated in the SSC. In addition, *Brachypodium stacei* and *Briza maxima* showed significant differences in boundary characteristics from the other species. It was clearly observed that the IR regions of *Brachypodium stacei* were contracted, resulting in the distribution of the *rps19* gene originally located in this region to the LSC. However, the IR region of *Briza maxima* expanded, wrapping the *rpl22* that should have been located in the LSC.

### Similarity Analysis of Chloroplast Genomes

**FIGURE 4:**
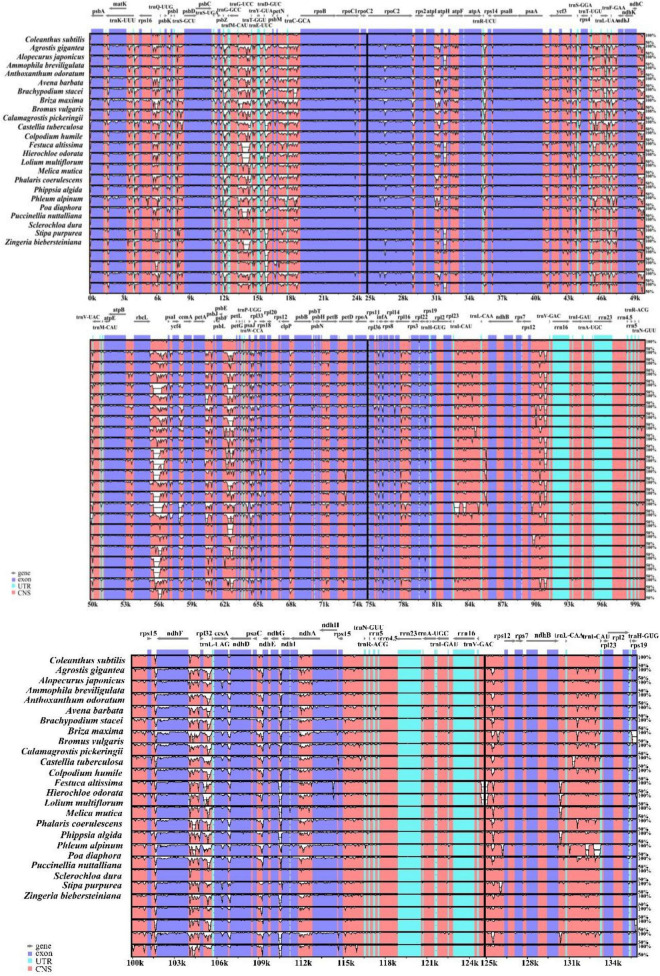
The Shuffle-LAGAN alignment was used in mVISTA to compare the contiguity of the chloroplast genomes of 24 species, with *C. subtilis* as the reference. The vertical scale in the figure indicates the degree of identity between 50% and 100%, while the horizontal scale shows the sequence information of the chloroplast genomes. Gray lines indicate gene direction, order and position.

Whole sequence alignment of the chloroplast genomes of 24 Pooideae species was performed to detect the differences that exist in their structures (Figure 4). The annotation of *Coleanthus subtilis* were used as a reference. The chloroplast genomes of these species were largely identical in terms of the number and arrangement of genes. However, some highly variable regions were still detected, such as *rbcL-psaI*, *psbE-petL*, *trnD-GUC-psbM*, *trnG-UCC-trnT-GGU*, *rpl32-trnL-UAG* and other intergenic regions. Overall, the non-coding regions showed a higher potential for variation compared to the coding regions. Although the protein-coding regions were relatively conserved, larger variants were observed in the *rpoC2*, *infA*, cemA and *matK* genes. Besides, variations were also presented in some genes located at the IR/SC boundary, such as *rps19* and *ndhF*. However, the rRNA and tRNA sequences were highly conserved, where genes such as *rrn16*, *rrn23*, *trnV-GAC*, and *trnR-ACG* were almost unchanged. At the same time, IR regions of these species were minimally altered and significantly more conserved than the two single-copy regions.

### Codon Usage Analysis

**FIGURE 5:**
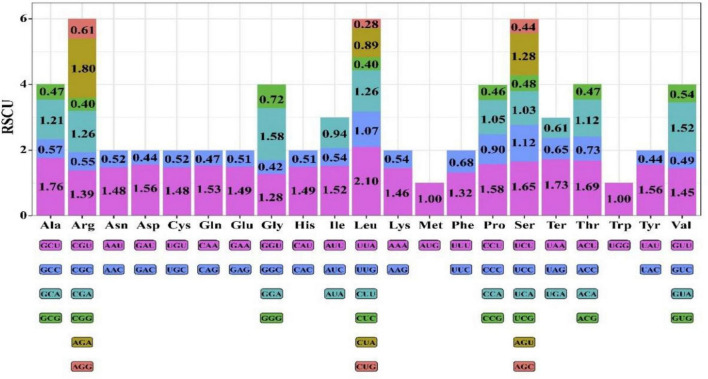
Relative synonymous codon usage (RSCU) values for amino acids and stop codons of the 76 protein-coding regions of *C*. *subtilis*. The colors of the histograms correspond to the colors of the codons.

There were 19838 codons eventually found in chloroplast genome of *Coleanthus subtilis*. Methionine and Tryptophan amino acids were encoded by a single codon, AUG and CGG, respectively. However, the remaining amino acids were encoded by two to six codons and showed a clear preference for codon usage (Figure 5). The most abundant amino acid in the *C*. *subtilis* was leucine 2135 (10.76%). Conversely, the least abundant amino acid was cysteine 218, which accounted for only 1.10% of the total. Meanwhile, among the six codons encoding leucine, UUA had the highest RSCU value of 2.10, which indicated that it had a high preference and was the most commonly used codon. Interestingly, most of the codons with RSCU values greater than 1 had A/U as the terminal codon, while those with C/G as the terminal codon usually had RSCU values less than 1.

**FIGURE 6:**
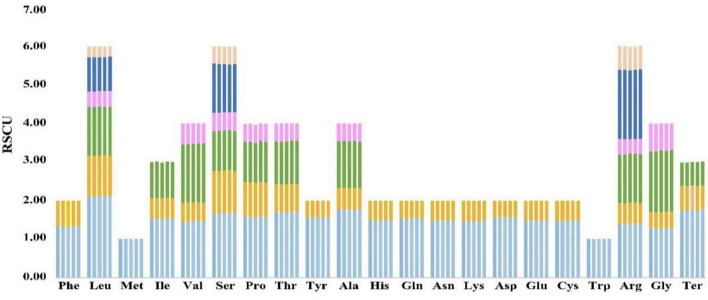
Comparative analysis plots of RSCU values for the five species. Each amino acid corresponds to five histograms, and their heights represent the RSCU value. The histogram from left to right is *Coleanthus subtilis*, *Phippsia algida*, *Puccinellia nuttalliana*, *Sclerochloa dura*, and *Zingeria biebersteiniana*.

The RSCU values of the five species were compared in order to understand the differences in their codon usage (Figure 6). For one amino acid, the sum of the RSCU values of all codons involved in its encoding was almost equal. Also, the RSCU values of the same codons were almost identical in these species, indicating that their codon usage habits were more stable and hardly change (Figure 6 and Supplementary Tables 4, 5).

### Repeat Analysis

**FIGURE 7:**
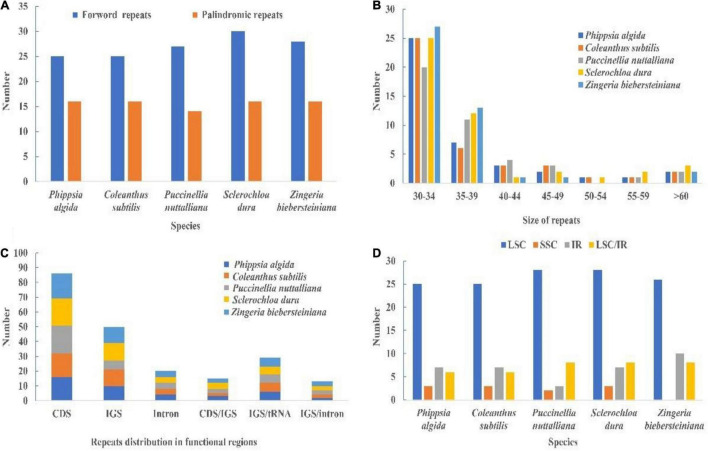
**(A)** Type of repeats in the whole chloroplast genomes of *C*. *subtilis* and its related species. **(B)** Size of repeats in the chloroplast genome of *C*. *subtilis* and its related species. **(C)** Distribution of repeats in functional regions of the plastid genome. **(D)** Distribution of repeats in regions of the chloroplast genomes. IR, inverted repeat; LSC, large single copy; SSC, small single copy; LSC/IR show those repeats for which one copy of the repeat exists in one region and a second copy exists in another region. CDS, protein-coding sequence; IGS, intergenic spacer region.

We detected only palindromic and forward repeats in chloroplast genomes of *Coleanthus subtilis* and its related species, where the proportion of forward repeats was higher than that of palindromic repeats (Figure 7A and Supplementary Table 6). Most of repeats were 30–34 bp in length and were mainly distributed in the LSC region (Figures 7B,D and Supplementary Tables 7, 8). Also, the CDS regions contained most of the repeats, followed by the IGS regions (Figure 7C and Supplementary Table 9). Some repeats were also shared between IGS, CDS, tRNA, and intron regions.

**FIGURE 8:**
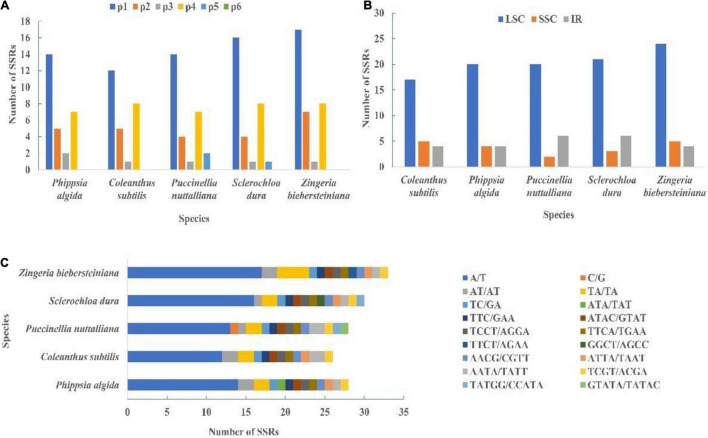
**(A)** The type of SSRs in the cp genome of *C*. *subtilis* and its related species. **(B)** The region of SSRs in the cp genome of *C*. *subtilis* and its related species. **(C)** The unit of SSRs in the cp genome of *C*. *subtilis* and its related species. IR, inverted repeat; LSC, large single copy; SSC, small single copy.

A total of 26 SSRs were detected in *C*. *subtilis*, while 28, 28, 30, and 33 microsatellites were found in *Phippsia algida*, *Puccinellia nuttalliana*, *Sclerochloa dura*, and *Zingeria biebersteiniana*, respectively (Figure 8A and Supplementary Table 10). These SSRs were classified into five types, namely Mono-, di-, tri-, tetra-, and penta-nucleotides repeats. The mono-nucleotide repeats accounted for 50.34% of the 145 microsatellites and were the most abundant SSR types in the five species, followed by tetra-nucleotide repeats (26.21%). Most of the microsatellites were distributed in the LSC region and consisted of A/T motifs (Figures 8B,C and Supplementary Tables 11, 12).

### Nucleotide Diversity (*Pi*) and Selection Pressure Analysis

**FIGURE 9:**
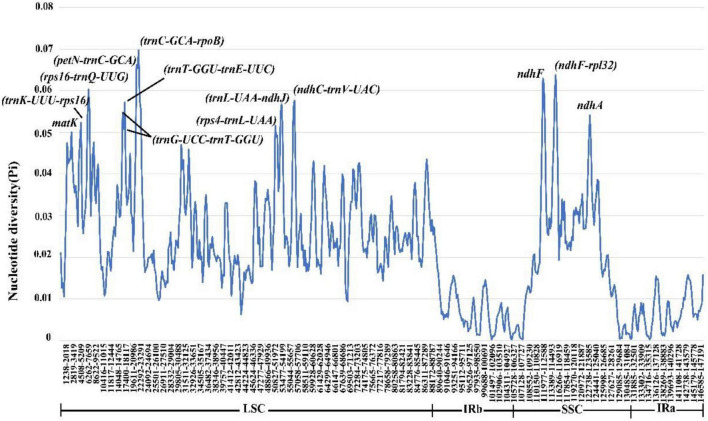
Nucleotide diversity of the chloroplast genomes of *C. subtilis* and its related species.

To comprehensively understand the sequence divergence of the chloroplast genomes of *Coleanthus subtilis* and its related species, we calculated *Pi* values for nucleotide diversity. *Pi* values fluctuated between 0 and 0.0697, with a mean value of 0.02172 (Figure 9). We identified 13 polymorphic regions (*matK*, *trnK-UUU/rps16*, *rps16/trnQ-UUG*, *trnG-UCC/trnT-GGU*, *trnT-GGU/trnE-UUC*, *petN/trnC-GCA*, *trnC-GCA/rpoB*, *rps4/trnL-UAA*, *trnL-UAA/ndhJ*, *ndhC/trnV-UAC*, *ndhF*, *ndhF/rpl32*, and *ndhA*) with nucleotide diversity >0.05, 10 of which were intergenic spacer regions and the remaining three were protein-coding regions. Meanwhile, no highly variable loci were detected in the IR regions and the nucleotide diversity values were significantly lower than those in the single copy regions (Figure 9 and Supplementary Table 13).

In this study, dN/dS values were calculated based on 76 CDS regions with site models in EasyCodeML. According to the M8 model, only the *atpF* gene possessed a significant positive site in the BEB approach (Table 3). Meanwhile, a total of 45 loci corresponding to 21 genes were identified in the NEB method, of which 13 genes (*atpA*, *atpF*, *atpI*, *ccsA*, *clpP*, *infA*, *ndhA*, ndhD, *ndhK*, *rbcL*, *rpoA*, *rps16*, and *rps3*) had a significant positive site. In addition, the *ndhF*, *psaA*, *psaB*, *psbC*, and *rpoC1* genes contained two significant positive selection loci, while the *cemA* and *matK* genes were detected with three and eight loci under positive selection, respectively. Moreover, the *rpoC2* gene was found to have the highest number of positive selection sites, including 11 significant positive sites.

**TABLE 3:** dN/dS ratios of the chloroplast genomes of *C. subtilis* and its related species.

M8	Gene	Region	Selected sites	Pr(*w* > 1)	Number of sites
Naive Empirical Bayes (NEB)	*atpA*	LSC	133 A	0.958*	1
	*atpF*	LSC	1247 A	1.000**	1
	*atpI*	LSC	1646 L	0.998**	1
	*ccsA*	SSC	1824 F	0.963*	1
	*cemA*	LSC	1989 L/2043 R/2092 F	0.969*/0.958*/0.986*	3
	*clpP*	LSC	2370 E	0.964*	1
	*infA*	LSC	2521 H	0.966*	1
	*matK*	LSC	2558 C/2599 S/2711 I/2713 I/2805 L/2807 L/2882 V/2937 Q	0.958*/0.954*/0.951*/0.956*/0.956*/0.965*/0.953*/0.970*	8
	*ndhA*	SSC	3322 G	0.954*	1
	*ndhD*	SSC	4425 I	0.995**	1
	*ndhF*	SSC	5107 S/5119 I	0.952*/0.951*	2
	*ndhK*	LSC	6446 K	0.959*	1
	*psaA*	LSC	7436 L/7917 V	0.954*/0.960*	2
	*psaB*	LSC	8195 S/8372 L	0.954*/0.950*	2
	*psbC*	LSC	10060 F/10233 F	0.951*/0.953*	2
	*rbcL*	LSC	11311 V	0.955*	1
	*rpoA*	LSC	12867 G	0.959*	1
	*rpoC1*	LSC	14684 G/14758 R	0.967*/0.959*	2
	*rpoC2*	LSC	15415 N/15426 I/15775 A/15836 F/15841 S/15860 K/15891 K/15907 F/16026 E/16204 Q/16260 A	0.965*/0.968*/0.998**/0.958*/0.953*/0.962*/0.962*/0.964*/0.964*/0.961*/0.965*	11
	*rps16*	LSC	16812 T	0.967*	1
	*rps3*	LSC	17399 E	0.955*	1
Bayes Empirical Bayes (BEB)	*atpF*	LSC	1247 A	0.974*	1

The level of significance is indicated by the number of “*”, where “*” represents significant, and “**” indicates highly significant.

### Phylogenetic Analysis

**FIGURE 10:**
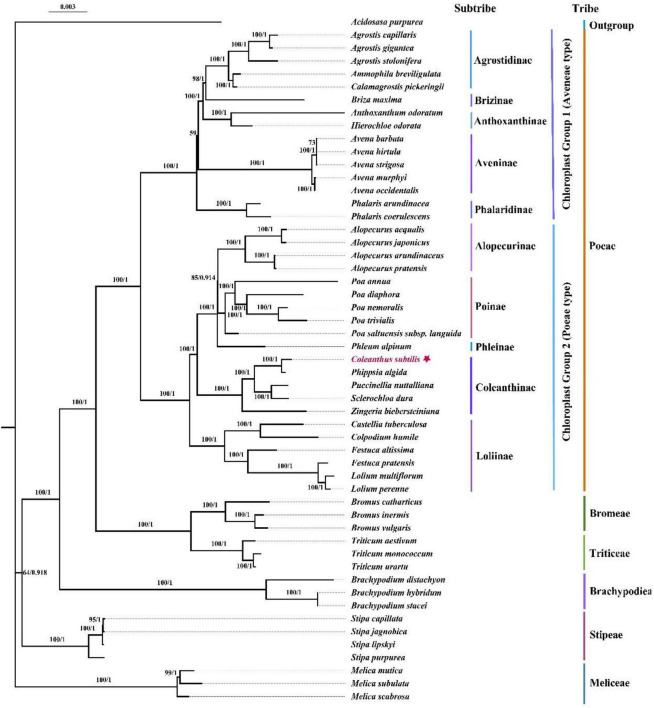
Phylogenetic trees were generated based on the 76 shared protein-coding sequences of 53 species using maximum likelihood (ML) and Bayesian (BI) methods. The ML tree and BI tree have a consistent topology. The ML bootstrap values/Bayesian posterior probabilities are displayed on the nodes. To make *Coleanthus subtilis* more visible, it was marked with a star.

In the current study, we utilized the protein-coding regions of chloroplast genomes for the first time to explore the phylogenetic position of *Coleanthus subtilis*. The topologies of the phylogenetic trees generated with maximum likelihood (ML) and Bayesian analysis (BI) were identical, with generally high branch bootstrap values and posterior probabilities. Based on consistent topologies, we showed the phylogenetic tree represented by the ML method (Figure 10). The 53 species representing 26 genera were divided into ten subtribes and six tribes. Among them, *Coleanthus* was placed in the big clade containing *Phippsia*, *Puccinellia*, *Sclerochloa*, and *Zingeria*, which were components of the subtribe Coleanthinae. In addition, *C*. *subtilis* formed a sister branch with the genus *Phippsia*, while this branch was also sister to other taxa of this subtribe (BS = 100, PP = 1). The genus *Colpodium* was nested in the subclade Loliinae and had a sister relationship with the genus *Castellia* (BS = 100, PP = 1).

## Discussion

### Plastome Comparison of *Coleanthus subtilis* and Other Species Within Pooideae

The chloroplast genome of *Coleanthus subtilis* exhibited a tetrad structure of 135915 bp in length, which is similar to the length and structural characteristics of cp genomes of other higher plants (Jansen et al., 2005; Daniell et al., 2016). We found that the GC content in the cp genome of Pooideae species was unevenly distributed, with the IR regions having a higher GC content than the two single copy regions. This may be attributed to the fact that four rRNA genes with high GC content were located in the IR regions, which supported the speculation of previous studies (Mardanov et al., 2008; Gao et al., 2009; Wanga et al., 2021). The *accD* gene has been lost within the cp genomes of Pooideae species, while *ycf1*, *ycf2*, *ycf15*, and *ycf68* were pseudogenes, which is a relatively common phenomenon in Poaceae (Huang et al., 2017). There is a correlation between gene loss and evolution, and some studies suggest that it may be an adaptive strategy with positive effects on survival and reproduction (Xu and Guo, 2020). In addition, we also found *trnL-UAA* gene loss in *Bromus vulgaris*. Pseudogenization of tRNA (*trnT-GGU*) has also been observed in the Asteraceae family (Abdullah et al., 2021a). Sixteen intron-containing genes were detected in 24 species in which introns of *rpoC1* and *clpP* genes were lost. Besides, the *trnK-UUU* has the longest intron that completely wraps the *matK* gene, a result that has been reported in other studies (Li X. et al., 2019; Souza et al., 2020). The *rpoC1* gene has been reported to contain introns in most land plants (Ohyama et al., 1986; Kugita et al., 2003). However, deletion of the *rpoC1* intron was observed in some angiosperm lineages, such as most Poaceae and some species of the families Fabaceae, Cactaceae, and Aizoaceae (Downie et al., 1996; Wallace and Cota, 1996; Huang et al., 2017). Our study on the subfamily Pooideae further confirm that the absence of the *rpoC1* intron is universal in the Poaceae. Similarly, the *clpP* gene usually contained two introns. Nevertheless, both introns have been lost in *Pinus* and some species from the genera *Oenothera*, *Silene*, and *Menodora* (Lee et al., 2007; Huang et al., 2017). Also, it was demonstrated that the loss of *clpP* introns were present in all Poaceae species (Guisinger et al., 2010), which was supported by our findings. This study revealed that genomic structure, gene content and total GC content were significantly similar or identical within 24 genera from Pooideae, which were consistent with the genus *Blumea* and the families Solanaceae, Malvaceae, and Araceae (Abdullah et al., 2020b,c, 2021b).

Length variation in the IR region of the chloroplast genome was a common phenomenon during the evolution of land plants, which has led to the formation of diverse boundary features (Yang et al., 2010; Wang et al., 2017; Ding et al., 2021). The study demonstrated that boundary genes in the species of the subfamily Pooideae were mainly *rpl22*, *rps19*, *rps15*, *ndhF*, *ndhH*, and *psbA*, which differ from the boundaries of *Clethra* and *Blumea* species (Abdullah et al., 2021b; Ding et al., 2021). In general, the subfamily Pooideae shared many similarities at the nodes, which further endorsed the idea that the boundary features were relatively stable among closely related species (Liu et al., 2018). This phenomenon has also been observed in the subfamily Asteroideae (Abdullah et al., 2021b). However, distinct junction characteristics also existed in related species, such as *Brachypodium stacei* and *Briza maxima*. The present study found that although both were species of the subfamily Pooideae, they formed different boundary features due to noticeable contraction or expansion of the IR Regions, respectively. The same findings were also noted in the genera *Pelargonium* and *Psilotum* (Chumley et al., 2006; Grewe et al., 2013; Sun et al., 2013).

The results of the mVISTA analysis showed that the coding regions were more conserved than the non-coding regions in the cp genomes of the subfamily Pooideae, and the two single copy regions showed higher variation potential than the IR regions. These two findings agreed with previous studies in other plant taxa (Gu et al., 2016; Xu et al., 2017; Alzahrani et al., 2020). We detected some highly variable non-coding regions, such as *rbcL-psaI*, *psbE-petL*, *trnD-GUC-psbM*, and *rpl32-trnL-UAG*. Despite the relative conservation of the protein-coding regions, variations were also observed in *rpoC2*, *infA*, *cemA*, and *matK* genes. The highly variable regions detected in this study were promising to be developed as specific DNA barcodes for the subfamily Pooideae, which has positive implications for the identification of species. In addition, the high GC content might be one of the reasons for less variation in tRNA sequences and IR regions, which further demonstrates the significance of GC content in maintaining sequence stability (Necsulea and Lobry, 2007; Kim et al., 2019).

The codon usage preference is closely related to gene expression and affects protein and mRNA levels in the genome (Zhou et al., 2013; Lyu and Liu, 2020). The most abundant amino acid in the *C*. *subtilis* was leucine 2135 (10.76%), which has also been frequently reported in the chloroplast genomes of other angiosperms (Jian et al., 2018; Somaratne et al., 2019). More interestingly, most codons ending in A/U have RSCU values greater than 1, while those ending in C/G are less than 1. This pattern also applies to the preference of codon usage in other plants (Wang et al., 2018; Liu X.Y. et al., 2020).

Oligonucleotide repeats are very common in plastid genome and are thought to be a proxy for identifying mutational hotspots (Ahmed et al., 2012; Lee et al., 2014; Abdullah et al., 2020a,d; Liu Q. et al., 2020). In the present study, we detected both forward and palindromic repeats, mostly distributed in the LSC region. Additionally, most of the repeats were 30–40 bp in length, which was similar to those found in other species (Chen et al., 2018; Li D.M. et al., 2019; Wu et al., 2020). Simple sequence repeats (SSRs) were often used as a molecular marker to explore population relationships and evolutionary history due to its polymorphism, co-dominance and reliability (Oliveira et al., 2006; Sonah et al., 2011; Gao et al., 2018). A total of five types of SSRs were detected in the cp genomes of *C*. *subtilis* and its related species, of which mono-nucleotide repeats were the most common. Similarly, the most abundant SSR type in the genus *Quercus* was also mono-nucleotide repeats (Yang et al., 2016). However, there are other possibilities, such as tri-nucleotide repeats occurring most frequently in *Urophysa* (Xie et al., 2018). Furthermore, this study not only found that most SSR types were mono-nucleotide repeats, but they had A/T preference. This phenomenon can also be observed in numerous other taxa (Wheeler et al., 2014; Munyao et al., 2020).

We identified 13 polymorphic regions (*matK*, *trnK-UUU/rps16*, *rps16/trnQ-UUG*, *trnG-UCC/trnT-GGU*, *trnT-GGU/trnE-UUC*, *petN/trnC-GCA*, *trnC-GCA/rpoB*, *rps4/trnL-UAA*, *trnL-UAA/ndhJ*, *ndhC/trnV-UAC*, *ndhF*, *ndhF/rpl32*, and *ndhA*) with nucleotide diversity >0.05, mainly located in the LSC region. In addition, the nucleotide diversity values within the IR regions were significantly lower than those in the single copy regions, which is consistent with the pattern found in previous studies (Li D.M. et al., 2019; Ding et al., 2021). The dN/dS analysis was regarded as one of the most popular and reliable measures to quantify selective pressure (Kryazhimskiy and Plotkin, 2008; Mugal et al., 2014). We performed a selection pressure analysis on different genera of the subfamily Pooideae, and the result indicated that there are some genes under positive selective pressure, which was crucial for understanding the evolutionary history of these genera. The positively selected genes identified were nearly identical to those previously reported for other species in the family Poaceae, and our findings further support the plausibility of these loci (Piot et al., 2018). Furthermore, these genes are associated with photosynthesis, self-expression and regulatory activity (Piot et al., 2018), which has a positive effect on understanding the mechanisms of selection pressure generation.

### Phylogenetic Analysis

In the current study, the 76 protein-coding regions of the chloroplast genome were used for the first time to explore the phylogenetic position of *Coleanthus subtilis*. The reconstructed phylogenetic tree divided the 53 species into ten subtribes and six tribes, which coincided with the broad framework of the Poaceae phylogeny (Soreng et al., 2017; Saarela et al., 2018; Tkach et al., 2020). Phylogenetic analysis strongly demonstrated that *C*. *subtilis* formed a sister branch with the genus *Phippsia* (BS = 100, PP = 1), which further justified the results of previous morphological treatments and phylogenetic studies based on chloroplast fragments (Tzvelev, 1976; Soreng et al., 2015; Gnutikov et al., 2020). Moreover, our data revealed that *Colpodium* was nested in the subtribe Loliinae and was particularly closely related to the genus *Castellia*, while *Zingeria* was located in the subtribe Coleanthinae (BS = 100, PP = 1). This finding differed from that of earlier studies and provided a new perspective on the relationships between *Colpodium*, *Zingeria* and Coleanthinae. Some previous studies suggested that the genera *Zingeria* and *Colpodium* are sister groups and rather distantly related to the subtribe Coleanthinae, forming a branch known as the two-chromosome grasses (Rodionov et al., 2008; Kim et al., 2009). At the same time, these two genera were considered as constituent members of Coleanthinae (Soreng et al., 2015). However, apart from the fact that *Zingeria* belongs to the subtribe Coleanthinae, our results do not support the previously reported relationship between *Colpodium*, *Zingeria* and Coleanthinae. This work will not only contribute to further insight into the phylogenetic position of *C*. *subtilis* and the composition of the subtribe Coleanthinae, but also provide valuable chloroplast genomic information for future exploration of the origin and differentiation between *C*. *subtilis* and its related species at the cp genome level.

## Conclusion

In this study, the complete chloroplast genome of *Coleanthus subtilis* was reported and comparative and phylogenetic analyses with its closely related species revealed, as well as differences in their genomic structure and composition. Although the chloroplast genome of *C. subtilis* is relatively conserved, 26 SSRs and 13 highly variable loci were detected, which could be developed as important genetic markers. The reconstructed phylogenetic tree further confirmed the sister relationship between *Coleanthus* and *Phippsia*, and also provided new insights into the relationship between *Coleanthus*, *Zingeria* and *Colpodium*. In addition, since *C. subtilis* is rare and legally protected, the genetic information is important for its breeding and conservation. Equally important, the mechanisms that lead to the unique distribution pattern of *C. subtilis* are unknown, which makes the species of great research value. Our results will enrich data and provide a useful reference for further research on the origin and distribution of *C. subtilis*.

## Data Availability Statement

The datasets presented in this study can be found in online repositories. The names of the repository/repositories and accession number(s) can be found in the article/Supplementary Material.

## Author Contributions

G-WH and X-ZC designed the topic. JR, JT, and XD participated in the sample collection. X-XZ was the first to discover *Coleanthus subtilis*, which inspired us to do research on it. And he assisted in the process of collecting samples. JR analyzed the chloroplast genome data and wrote the manuscript. JT designed the protocol and conducted the experiment. HJ, S-XD, J-XY, and L-LC provided guidance and assistance during the analysis of the data. Also, FM and VW provided valuable comments in writing the article. All authors contributed to this study and approved the final submitted manuscript.

## Conflict of Interest

The authors declare that the research was conducted in the absence of any commercial or financial relationships that could be construed as a potential conflict of interest.

## Publisher’s Note

All claims expressed in this article are solely those of the authors and do not necessarily represent those of their affiliated organizations, or those of the publisher, the editors and the reviewers. Any product that may be evaluated in this article, or claim that may be made by its manufacturer, is not guaranteed or endorsed by the publisher.

## Footnotes

1
https://www.forestry.gov.cn/

2
https://chlorobox.mpimp-golm.mpg.de/geseq.html

3
https://chlorobox.mpimp-golm.mpg.de/OGDraw.html

4
https://bibiserv.cebitec.uni-bielefeld.de/reputer

5
https://www.figtreeasia.com/
