# Supplementary material for: Comparative and Phylogenetic Analysis Based on the Chloroplast Genome of Coleanthus subtilis (Tratt.) Seidel, a Protected Rare Species of Monotypic Genus
Source: Front Plant Sci. 2022 Feb 24;13:828467. doi: 10.3389/fpls.2022.828467 (PMC8908325; doi:10.3389/fpls.2022.828467)
Supplement: Supplementary file 1 [file Data_Sheet_1.zip › Supplementary Table/Supplementary Table 1.docx]

**Supplementary Table 1.** NCBI accession number of 53 chloroplast genomes.

| **Number** | **Species** | **Accession** |
| --- | --- | --- |
| 1 | *Coleanthus subtilis* | OL692806 |
| 2 | *Phippsia algida* | MT094324 |
| 3 | *Colpodium humile* | MT113379 |
| 4 | *Zingeria biebersteiniana* | NC_037170 |
| 5 | *Puccinellia nuttalliana* | NC_027485 |
| 6 | *Sclerochloa dura* | MT094329 |
| 7 | *Poa saltuensis subsp. languida* | MT094326 |
| 8 | *Poa diaphora* | MT094306 |
| 9 | *Poa trivialis* | NC_036975 |
| 10 | *Poa nemoralis* | NC_036974 |
| 11 | *Poa annua* | NC_036973 |
| 12 | *Phleum alpinum* | NC_027482 |
| 13 | *Alopecurus japonicus* | NC_047229 |
| 14 | *Alopecurus aequalis* | NC_047228 |
| 15 | *Alopecurus pratensis* | MW309817 |
| 16 | *Alopecurus arundinaceus* | NC_037163 |
| 17 | *Avena hirtula* | NC_050395 |
| 18 | *Avena occidentalis* | NC_044175 |
| 19 | *Avena murphyi* | NC_044174 |
| 20 | *Avena barbata* | NC_044173 |
| 21 | *Avena strigosa* | NC_044171 |
| 22 | *Briza maxima* | NC_027471 |
| 23 | *Brachypodium distachyon* | NC_011032 |
| 24 | *Brachypodium hybridum* | NC_036836 |
| 25 | *Brachypodium stacei* | NC_036837 |
| 26 | *Bromus catharticus* | NC_054212 |
| 27 | *Bromus vulgaris* | NC_027472 |
| 28 | *Bromus inermis* | MW861351 |
| 29 | *Lolium perenne* | NC_009950 |
| 30 | *Lolium multiflorum* | NC_019651 |
| 31 | *Melica scabrosa* | NC_050212 |
| 32 | *Melica subulata* | NC_027478 |
| 33 | *Melica mutica* | NC_027477 |
| 34 | *Phalaris coerulescens* | MT094323 |
| 35 | *Phalaris arundinacea* | NC_027481 |
| 36 | *Hierochloe odorata* | NC_027475 |
| 37 | *Stipa lipskyi* | NC_028444 |
| 38 | *Stipa purpurea* | NC_029390 |
| 39 | *Stipa capillata* | MZ145043 |
| 40 | *Stipa jagnobica* | NC_037029 |
| 41 | *Anthoxanthum odoratum* | NC_027467 |
| 42 | *Castellia tuberculosa* | NC_042703 |
| 43 | *Ammophila breviligulata* | NC_027465 |
| 44 | *Agrostis gigantea* | NC_037162 |
| 45 | *Agrostis capillaris* | MW143240 |
| 46 | *Agrostis stolonifera* | NC_008591 |
| 47 | *Calamagrostis pickeringii* | NC_050417 |
| 48 | *Triticum aestivum* | KC912694 |
| 49 | *Triticum urartu* | NC_021762 |
| 50 | *Triticum monococcum* | NC_021760 |
| 51 | *Festuca pratensis* | NC_019650 |
| 52 | *Festuca altissima* | NC_019648 |
| 53 | *Acidosasa purpurea* | NC_015820 |
