# Supplementary material for: Comparative and Phylogenetic Analysis Based on the Chloroplast Genome of Coleanthus subtilis (Tratt.) Seidel, a Protected Rare Species of Monotypic Genus
Source: Front Plant Sci. 2022 Feb 24;13:828467. doi: 10.3389/fpls.2022.828467 (PMC8908325; doi:10.3389/fpls.2022.828467)
Supplement: Supplementary file 1 [file Data_Sheet_1.zip › Supplementary Table/Supplementary Table 10.docx]

| **SSR type** | **Species** | | | | | |
| --- | --- | --- | --- | --- | --- | --- |
|  | ***Phippsia algida*** | ***Coleanthus subtilis*** | ***Puccinellia nuttalliana*** | ***Sclerochloa dura*** | ***Zingeria biebersteiniana*** | |
| p1 | 14 | 12 | 14 | 16 | | 17 |
| p2 | 5 | 5 | 4 | 4 | | 7 |
| p3 | 2 | 1 | 1 | 1 | | 1 |
| p4 | 7 | 8 | 7 | 8 | | 8 |
| p5 | 0 | 0 | 2 | 1 | | 0 |
| p6 | 0 | 0 | 0 | 0 | | 0 |
| total | 28 | 26 | 28 | 30 | | 33 |

**Supplementary Table 10.** The type of SSRs in the cp genome of *C. subtilis* and its related species
