# Supplementary material for: Comparative and Phylogenetic Analysis Based on the Chloroplast Genome of Coleanthus subtilis (Tratt.) Seidel, a Protected Rare Species of Monotypic Genus
Source: Front Plant Sci. 2022 Feb 24;13:828467. doi: 10.3389/fpls.2022.828467 (PMC8908325; doi:10.3389/fpls.2022.828467)
Supplement: Supplementary file 1 [file Data_Sheet_1.zip › Supplementary Table/Supplementary Table 11.docx]

| **Region of microsatelites** | **Species** | | | | |
| --- | --- | --- | --- | --- | --- |
|  | ***Coleanthus subtilis*** | ***Phippsia algida*** | ***Puccinellia nuttalliana*** | ***Sclerochloa dura*** | ***Zingeria biebersteiniana*** |
| LSC | 17 | 20 | 20 | 21 | 24 |
| SSC | 5 | 4 | 2 | 3 | 5 |
| IR | 4 | 4 | 6 | 6 | 4 |

**Supplementary Table 11.** The region of SSRs in the cp genome of *C. subtilis* and its related species
