# Supplementary material for: Comparative and Phylogenetic Analysis Based on the Chloroplast Genome of Coleanthus subtilis (Tratt.) Seidel, a Protected Rare Species of Monotypic Genus
Source: Front Plant Sci. 2022 Feb 24;13:828467. doi: 10.3389/fpls.2022.828467 (PMC8908325; doi:10.3389/fpls.2022.828467)
Supplement: Supplementary file 1 [file Data_Sheet_1.zip › Supplementary Table/Supplementary Table 12.docx]

| **SSR units** | **Species** | | | | |
| --- | --- | --- | --- | --- | --- |
|  | ***Phippsia algida*** | ***Coleanthus subtilis*** | ***Puccinellia nuttalliana*** | ***Sclerochloa dura*** | ***Zingeria biebersteiniana*** |
| A/T | 14 | 12 | 13 | 16 | 17 |
| C/G | 0 | 0 | 1 | 0 | 0 |
| AT/AT | 2 | 2 | 1 | 1 | 2 |
| TA/TA | 2 | 2 | 2 | 2 | 4 |
| TC/GA | 1 | 1 | 1 | 1 | 1 |
| ATA/TAT | 1 | 0 | 0 | 0 | 0 |
| TTC/GAA | 1 | 1 | 1 | 1 | 1 |
| ATAC/GTAT | 1 | 1 | 1 | 1 | 1 |
| TCCT/AGGA | 1 | 1 | 1 | 1 | 1 |
| TTCA/TGAA | 1 | 1 | 1 | 1 | 1 |
| TTCT/AGAA | 0 | 0 | 0 | 0 | 1 |
| GGCT/AGCC | 0 | 0 | 0 | 1 | 0 |
| AACG/CGTT | 1 | 1 | 1 | 1 | 1 |
| ATTA/TAAT | 1 | 1 | 0 | 1 | 1 |
| AATA/TATT | 1 | 2 | 2 | 1 | 1 |
| TCGT/ACGA | 1 | 1 | 1 | 1 | 1 |
| TATGG/CCATA | 0 | 0 | 1 | 1 | 0 |
| GTATA/TATAC | 0 | 0 | 1 | 0 | 0 |

**Supplementary Table 12.** The unit of SSRs in the cp genome of *C. subtilis* and its related species
