# Supplementary material for: Comparative and Phylogenetic Analysis Based on the Chloroplast Genome of Coleanthus subtilis (Tratt.) Seidel, a Protected Rare Species of Monotypic Genus
Source: Front Plant Sci. 2022 Feb 24;13:828467. doi: 10.3389/fpls.2022.828467 (PMC8908325; doi:10.3389/fpls.2022.828467)
Supplement: Supplementary file 1 [file Data_Sheet_1.zip › Supplementary Table/Supplementary Table 13.docx]

| **Region** | **Nucleotide diversity** | **Chloroplast region** |
| --- | --- | --- |
| *matK* | 0.05007 | LSC |
| *trnK-UUU-rps16* | 0.05231 | LSC |
| *trnK-UUU-rps16* | 0.05339 | LSC |
| *rps16-trnQ-UUG* | 0.06033 | LSC |
| *trnQ-UUG-psbK* | 0.05527 | LSC |
| *trnG-UCC-trnT-GGU* | 0.05463 | LSC |
| *trnG-UCC-trnT-GGU* | 0.05084 | LSC |
| *trnT-GGU-trnE-UUC* | 0.05724 | LSC |
| *petN-trnC-GCA* | 0.06618 | LSC |
| *petN-trnC-GCA* | 0.06544 | LSC |
| *trnC-GCA-rpoB* | 0.0697 | LSC |
| *trnC-GCA-rpoB* | 0.05767 | LSC |
| *trnC-GCA-rpoB* | 0.05559 | LSC |
| *rps4-trnT-UGU* | 0.0517 | LSC |
| *trnL-UAA-ndhJ* | 0.05441 | LSC |
| *trnL-UAA-ndhJ* | 0.05683 | LSC |
| *ndhC-trnV-UAC* | 0.05562 | LSC |
| *ndhC-trnV-UAC* | 0.05768 | LSC |
| *ndhF* | 0.06298 | SSC |
| *ndhF* | 0.0612 | SSC |
| *ndhF* | 0.05061 | SSC |
| *ndhF-rpl32* | 0.05954 | SSC |
| *ndhF-rpl32* | 0.06374 | *SSC* |
| *ndhF-rpl32* | 0.0539 | *SSC* |
| *ndhA* | 0.05405 | *SSC* |

**Supplementary Table 13.** Nucleotide diversity of the chloroplast genomes of *C. subtilis* and its related species.
