# Supplementary material for: Comparative and Phylogenetic Analysis Based on the Chloroplast Genome of Coleanthus subtilis (Tratt.) Seidel, a Protected Rare Species of Monotypic Genus
Source: Front Plant Sci. 2022 Feb 24;13:828467. doi: 10.3389/fpls.2022.828467 (PMC8908325; doi:10.3389/fpls.2022.828467)
Supplement: Supplementary file 1 [file Data_Sheet_1.zip › Supplementary Table/Supplementary Table 2.docx]

**Supplementary Table 2.** The gene number of the cp genome of *C. subtilis* in four regions.

| **Region** | **Number of CDS** | **Number of tRNA** | **Number of rRNA** | **Total** |
| --- | --- | --- | --- | --- |
| LSC | 59 | 23 | 0 | 82 |
| SSC | 10 | 1 | 0 | 11 |
| IRA | 7 | 7 | 4 | 18 |
| IRB | 7 | 7 | 4 | 18 |
