# Supplementary material for: Comparative and Phylogenetic Analysis Based on the Chloroplast Genome of Coleanthus subtilis (Tratt.) Seidel, a Protected Rare Species of Monotypic Genus
Source: Front Plant Sci. 2022 Feb 24;13:828467. doi: 10.3389/fpls.2022.828467 (PMC8908325; doi:10.3389/fpls.2022.828467)
Supplement: Supplementary file 1 [file Data_Sheet_1.zip › Supplementary Table/Supplementary Table 3.docx]

**Supplementary Table 3. Comparison of the kinds and length of introns among 24 chloroplast genome sequences.**

| **Species** | **Genes with one Intron** | | | | | | | | | | | | | | | **Genes with two Introns** | |
| --- | --- | --- | --- | --- | --- | --- | --- | --- | --- | --- | --- | --- | --- | --- | --- | --- | --- |
|  | *atpF* | *ndhA* | *ndhB* | *petB* | *petD* | *rpl2* | *rpl16* | *rps12* | *rps16* | *trnI-GAU* | *trnA-UGC* | *trnG-UCC* | *trnV-UAC* | *trnL-UAA* | *trnK-UUU* | *ycf3* | |
| *Coleanthus subtilis* | 814bp | 1028bp | 712bp | 767bp | 743bp | 663bp | 895bp | 540bp | 828bp | 806bp | 811bp | 676bp | 604bp | 537bp | 2480bp | 774bp | 726bp |
| *Phippsiaalgida* | 814bp | 1029bp | 712bp | 761bp | 743bp | 663bp | 888bp | 540bp | 828bp | 806bp | 811bp | 676bp | 602bp | 537bp | 2490bp | 774bp | 726bp |
| *Puccinellianuttalliana* | 809bp | 1030bp | 712bp | 767bp | 743bp | 663bp | 901bp | 540bp | 824bp | 806bp | 811bp | 676bp | 602bp | 537bp | 2511bp | 767bp | 728bp |
| *Sclerochloa dura* | 813bp | 1024bp | 712bp | 763bp | 743bp | 663bp | 901bp | 540bp | 826bp | 806bp | 811bp | 676bp | 602bp | 535bp | 2510bp | 756bp | 731bp |
| *Zingeriabiebersteiniana* | 813bp | 1025bp | 712bp | 756bp | 738bp | 663bp | 903bp | 540bp | 835bp | 805bp | 811bp | 676bp | 602bp | 542bp | 2522bp | 755bp | 717bp |
| *Agrostis gigantea* | 838bp | 1040bp | 712bp | 764bp | 741bp | 663bp | 1048bp | 540bp | 837bp | 805bp | 811bp | 675bp | 596bp | 419bp | 2478bp | 754bp | 728bp |
| *Alopecurus japonicus* | 824bp | 1018bp | 713bp | 780bp | 744bp | 663bp | 896bp | 540bp | 827bp | 806bp | 811bp | 678bp | 604bp | 491bp | 2506bp | 755bp | 730bp |
| *Ammophilabreviligulata* | 833bp | 1024bp | 712bp | 760bp | 741bp | 663bp | 1046bp | 540bp | 833bp | 805bp | 811bp | 677bp | 596bp | 419bp | 2475bp | 753bp | 731bp |
| *Anthoxanthum odoratum* | 834bp | 1021bp | 712bp | 773bp | 740bp | 663bp | 1053bp | 540bp | 833bp | 806bp | 811bp | 681bp | 606bp | 545bp | 2469bp | 752bp | 736bp |
| *Avenabarbata* | 825bp | 1019bp | 712bp | 759bp | 741bp | 663bp | 1048bp | 540bp | 827bp | 807bp | 811bp | 677bp | 596bp | 331bp | 2436bp | 759bp | 725bp |
| *Brachypodiumstacei* | 822bp | 1047bp | 716bp | 812bp | 756bp | 667bp | 1055bp | 540bp | 824bp | 805bp | 811bp | 684bp | 599bp | 538bp | 2496bp | 739bp | 726bp |
| *Briza maxima* | 820bp | 1023bp | 712bp | 768bp | 747bp | 663bp | 1000bp | 540bp | 838bp | 806bp | 811bp | 685bp | 596bp | 418bp | 2502bp | 744bp | 743bp |
| *Bromus vulgaris* | 817bp | 1035bp | 712bp | 751bp | 764bp | 663bp | 1055bp | 544bp | 836bp | 807bp | 811bp | 682bp | 598bp | 537bp | 2483bp | 765bp | 726bp |
| *Calamagrostis pickeringii* | 833bp | 1024bp | 712bp | 765bp | 741bp | 663bp | 1045bp | 540bp | 833bp | 805bp | 811bp | 677bp | 596bp | 419bp | 2475bp | 746bp | 728bp |
| *Castelliatuberculosa* | 818bp | 1036bp | 719bp | 752bp | 742bp | 663bp | 909bp | 540bp | 827bp | 806bp | 811bp | 675bp | 602bp | 545bp | 2490bp | 749bp | 707bp |
| *Colpodiumhumile* | 821bp | 1003bp | 719bp | 751bp | 737bp | 663bp | 896bp | 540bp | 833bp | 806bp | 811bp | 675bp | 604bp | 536bp | 2495bp | 751bp | 717bp |
| *Festuca altissima* | 806bp | 1031bp | 712bp | 752bp | 676bp | 663bp | 848bp | 540bp | 827bp | 801bp | 811bp | 676bp | 602bp | 543bp | 2534bp | 747bp | 723bp |
| *Hierochloe odorata* | 836bp | 1007bp | 712bp | 760bp | 745bp | 663bp | 1047bp | 540bp | 833bp | 805bp | 811bp | 681bp | 596bp | 537bp | 2467bp | 739bp | 732bp |
| *Lolium multiflorum* | 832bp | 1015bp | 715bp | 747bp | 677bp | 663bp | 861bp | 540bp | 836bp | 802bp | 811bp | 678bp | 603bp | 550bp | 2530bp | 752bp | 717bp |
| *Melicamutica* | 828bp | 1007bp | 714bp | 801bp | 743bp | 663bp | 1008bp | 534bp | 796bp | 806bp | 811bp | 674bp | 598bp | 544bp | 2515bp | 739bp | 721bp |
| *Phalaris coerulescens* | 832bp | 1028bp | 712bp | 765bp | 741bp | 663bp | 1060bp | 540bp | 831bp | 806bp | 811bp | 676bp | 596bp | 549bp | 2484bp | 740bp | 728bp |
| *Phleum alpinum* | 821bp | 1039bp | 712bp | 754bp | 738bp | 663bp | 894bp | 540bp | 827bp | 806bp | 811bp | 682bp | 602bp | 538bp | 2497bp | 755bp | 727bp |
| *Poa diaphora* | 812bp | 1038bp | 712bp | 759bp | 743bp | 663bp | 907bp | 540bp | 828bp | 801bp | 811bp | 680bp | 603bp | 538bp | 2501bp | 744bp | 720bp |
| *Stipa purpurea* | 837bp | 1033bp | 712bp | 815bp | 750bp | 663bp | 1034bp | 540bp | 818bp | 807bp | 811bp | 673bp | 597bp | 526bp | 2499bp | 740bp | 713bp |
