# Supplementary material for: Comparative and Phylogenetic Analysis Based on the Chloroplast Genome of Coleanthus subtilis (Tratt.) Seidel, a Protected Rare Species of Monotypic Genus
Source: Front Plant Sci. 2022 Feb 24;13:828467. doi: 10.3389/fpls.2022.828467 (PMC8908325; doi:10.3389/fpls.2022.828467)
Supplement: Supplementary file 1 [file Data_Sheet_1.zip › Supplementary Table/Supplementary Table 4.docx]

**Supplementary Table 4**. RSCU values of *Coleanthus subtilis* and its related species.

| **Amio acid** | **codon** | **RSCU** | | | | |
| --- | --- | --- | --- | --- | --- | --- |
|  |  | ***Coleanthus subtilis*** | ***Phippsia algida*** | ***Puccinellia nuttalliana*** | ***Sclerochloa dura*** | ***Zingeria biebersteiniana*** |
| Phe | UUU | 1.32 | 1.32 | 1.32 | 1.32 | 1.34 |
|  | UUC | 0.68 | 0.68 | 0.68 | 0.68 | 0.66 |
| Leu | UUA | 2.1 | 2.11 | 2.12 | 2.11 | 2.13 |
|  | CUU | 1.26 | 1.26 | 1.27 | 1.26 | 1.26 |
|  | CUC | 0.4 | 0.4 | 0.41 | 0.41 | 0.42 |
|  | CUA | 0.89 | 0.89 | 0.87 | 0.87 | 0.89 |
|  | CUG | 0.28 | 0.28 | 0.28 | 0.28 | 0.27 |
|  | UUG | 1.07 | 1.06 | 1.05 | 1.07 | 1.04 |
| Ser | UCU | 1.65 | 1.66 | 1.67 | 1.69 | 1.67 |
|  | UCC | 1.12 | 1.11 | 1.11 | 1.11 | 1.1 |
|  | UCA | 1.03 | 1.04 | 1.04 | 1.03 | 1.03 |
|  | AGU | 1.28 | 1.27 | 1.26 | 1.25 | 1.26 |
|  | AGC | 0.44 | 0.45 | 0.45 | 0.46 | 0.45 |
|  | UCG | 0.48 | 0.47 | 0.47 | 0.46 | 0.49 |
| Tyr | UAU | 1.56 | 1.55 | 1.57 | 1.56 | 1.56 |
|  | UAC | 0.44 | 0.45 | 0.43 | 0.44 | 0.44 |
| Ter | UAA | 1.73 | 1.73 | 1.75 | 1.72 | 1.79 |
|  | UGA | 0.61 | 0.61 | 0.61 | 0.62 | 0.61 |
|  | UAG | 0.65 | 0.65 | 0.64 | 0.66 | 0.61 |
| Cys | UGU | 1.48 | 1.48 | 1.47 | 1.47 | 1.47 |
|  | UGC | 0.52 | 0.52 | 0.53 | 0.53 | 0.53 |
| Trp | UGG | 1 | 1 | 1 | 1 | 1 |
| Pro | CCU | 1.58 | 1.58 | 1.56 | 1.59 | 1.57 |
|  | CCC | 0.9 | 0.9 | 0.9 | 0.9 | 0.9 |
|  | CCA | 1.05 | 1.05 | 1.04 | 1.06 | 1.06 |
|  | CCG | 0.46 | 0.46 | 0.46 | 0.45 | 0.46 |
| His | CAU | 1.49 | 1.49 | 1.48 | 1.49 | 1.48 |
|  | CAC | 0.51 | 0.51 | 0.52 | 0.51 | 0.52 |
| Gln | CAA | 1.53 | 1.53 | 1.54 | 1.54 | 1.52 |
|  | CAG | 0.47 | 0.47 | 0.46 | 0.46 | 0.48 |
| Arg | CGU | 1.39 | 1.39 | 1.41 | 1.39 | 1.4 |
|  | CGC | 0.55 | 0.54 | 0.54 | 0.56 | 0.53 |
|  | CGA | 1.26 | 1.27 | 1.27 | 1.27 | 1.28 |
|  | AGA | 1.8 | 1.8 | 1.78 | 1.8 | 1.81 |
|  | AGG | 0.61 | 0.61 | 0.61 | 0.6 | 0.6 |
|  | CGG | 0.4 | 0.4 | 0.38 | 0.38 | 0.39 |
| Ile | AUU | 1.52 | 1.53 | 1.52 | 1.54 | 1.53 |
|  | AUC | 0.54 | 0.54 | 0.54 | 0.54 | 0.54 |
|  | AUA | 0.94 | 0.94 | 0.93 | 0.93 | 0.93 |
| Met | AUG | 1 | 1 | 1 | 1 | 1 |
| Thr | ACU | 1.69 | 1.69 | 1.69 | 1.7 | 1.7 |
|  | ACC | 0.73 | 0.73 | 0.73 | 0.73 | 0.72 |
|  | ACA | 1.12 | 1.11 | 1.12 | 1.12 | 1.13 |
|  | ACG | 0.47 | 0.47 | 0.46 | 0.45 | 0.45 |
| Asn | AAU | 1.48 | 1.49 | 1.48 | 1.47 | 1.48 |
|  | AAC | 0.52 | 0.51 | 0.52 | 0.53 | 0.52 |
| Lys | AAA | 1.46 | 1.46 | 1.46 | 1.46 | 1.47 |
|  | AAG | 0.54 | 0.54 | 0.54 | 0.54 | 0.53 |
| Val | GUU | 1.45 | 1.45 | 1.47 | 1.47 | 1.47 |
|  | GUC | 0.49 | 0.5 | 0.48 | 0.48 | 0.47 |
|  | GUA | 1.52 | 1.52 | 1.52 | 1.53 | 1.54 |
|  | GUG | 0.54 | 0.53 | 0.53 | 0.52 | 0.53 |
| Ala | GCU | 1.76 | 1.76 | 1.76 | 1.76 | 1.77 |
|  | GCC | 0.57 | 0.56 | 0.56 | 0.56 | 0.55 |
|  | GCA | 1.21 | 1.23 | 1.22 | 1.23 | 1.21 |
|  | GCG | 0.47 | 0.45 | 0.45 | 0.45 | 0.47 |
| Asp | GAU | 1.56 | 1.56 | 1.57 | 1.57 | 1.55 |
|  | GAC | 0.44 | 0.44 | 0.43 | 0.43 | 0.45 |
| Glu | GAA | 1.49 | 1.49 | 1.49 | 1.48 | 1.48 |
|  | GAG | 0.51 | 0.51 | 0.51 | 0.52 | 0.52 |
| Gly | GGU | 1.28 | 1.28 | 1.27 | 1.28 | 1.3 |
|  | GGC | 0.42 | 0.41 | 0.43 | 0.42 | 0.42 |
|  | GGA | 1.58 | 1.59 | 1.6 | 1.59 | 1.6 |
|  | GGG | 0.72 | 0.71 | 0.71 | 0.71 | 0.69 |
