# Supplementary material for: Comparative and Phylogenetic Analysis Based on the Chloroplast Genome of Coleanthus subtilis (Tratt.) Seidel, a Protected Rare Species of Monotypic Genus
Source: Front Plant Sci. 2022 Feb 24;13:828467. doi: 10.3389/fpls.2022.828467 (PMC8908325; doi:10.3389/fpls.2022.828467)
Supplement: Supplementary file 1 [file Data_Sheet_1.zip › Supplementary Table/Supplementary Table 5.docx]

**Supplementary Table 5.** Comparison of the relative synonymous codon usage (RSCU) among five chloroplast genomes.

| **Amino**  **acid types** | ***Coleanthus***  ***subtilis*** | ***Phippsia***  ***algida*** | ***Puccinellia***  ***nuttalliana*** | ***Sclerochloa***  ***dura*** | ***Zingeria***  ***biebersteiniana*** |
| --- | --- | --- | --- | --- | --- |
| Phe(F) | 2.00 | 2.00 | 2.00 | 2.00 | 2.00 |
| leu(L) | 6.00 | 6.00 | 6.00 | 6.00 | 6.01 |
| Ile(I) | 3.00 | 3.01 | 2.99 | 3.01 | 3.00 |
| Met(M) | 1.00 | 1.00 | 1.00 | 1.00 | 1.00 |
| Val(V) | 4.00 | 4.00 | 4.00 | 4.00 | 4.01 |
| Ser(S) | 6.00 | 6.00 | 6.00 | 6.00 | 6.00 |
| Pro(P) | 3.99 | 3.99 | 3.96 | 4.00 | 3.99 |
| Thr(T) | 4.01 | 4.00 | 4.00 | 4.00 | 4.00 |
| Ala(A) | 4.01 | 4.00 | 3.99 | 4.00 | 4.00 |
| Tyr(Y) | 2.00 | 2.00 | 2.00 | 2.00 | 2.00 |
| His(H) | 2.00 | 2.00 | 2.00 | 2.00 | 2.00 |
| Gln(Q) | 2.00 | 2.00 | 2.00 | 2.00 | 2.00 |
| Asn(N) | 2.00 | 2.00 | 2.00 | 2.00 | 2.00 |
| Lys(K) | 2.00 | 2.00 | 2.00 | 2.00 | 2.00 |
| Asp(D) | 2.00 | 2.00 | 2.00 | 2.00 | 2.00 |
| Glu(E) | 2.00 | 2.00 | 2.00 | 2.00 | 2.00 |
| Cys(C) | 2.00 | 2.00 | 2.00 | 2.00 | 2.00 |
| Trp(W) | 1.00 | 1.00 | 1.00 | 1.00 | 1.00 |
| Arg(R) | 6.01 | 6.01 | 5.99 | 6.00 | 6.01 |
| Gly(G) | 4.00 | 3.99 | 4.01 | 4.00 | 4.01 |
| Ter (*) | 2.99 | 2.99 | 3.00 | 3.00 | 3.01 |
