# Supplementary material for: Comparative and Phylogenetic Analysis Based on the Chloroplast Genome of Coleanthus subtilis (Tratt.) Seidel, a Protected Rare Species of Monotypic Genus
Source: Front Plant Sci. 2022 Feb 24;13:828467. doi: 10.3389/fpls.2022.828467 (PMC8908325; doi:10.3389/fpls.2022.828467)
Supplement: Supplementary file 1 [file Data_Sheet_1.zip › Supplementary Table/Supplementary Table 6.docx]

| **Species** | **Type of repeats** | |
| --- | --- | --- |
|  | Forword | Palindromic |
| *Phippsia algida* | 25 | 16 |
| *Coleanthus subtilis* | 25 | 16 |
| *Puccinellia nuttalliana* | 27 | 14 |
| *Sclerochloa dura* | 30 | 16 |
| *Zingeria biebersteiniana* | 28 | 16 |

**Supplementary Table 6.** Type of repeats in the whole chloroplast genomes of *C*. *subtilis* and its related species.
