# Supplementary material for: Comparative and Phylogenetic Analysis Based on the Chloroplast Genome of Coleanthus subtilis (Tratt.) Seidel, a Protected Rare Species of Monotypic Genus
Source: Front Plant Sci. 2022 Feb 24;13:828467. doi: 10.3389/fpls.2022.828467 (PMC8908325; doi:10.3389/fpls.2022.828467)
Supplement: Supplementary file 1 [file Data_Sheet_1.zip › Supplementary Table/Supplementary Table 7.docx]

| **Size of repeats** | **Species** | | | | |
| --- | --- | --- | --- | --- | --- |
|  | *Phippsia algida* | *Coleanthus subtilis* | *Puccinellia nuttalliana* | *Sclerochloa dura* | *Zingeria biebersteiniana* |
| 30-34 | 25 | 25 | 20 | 25 | 27 |
| 35-39 | 7 | 6 | 11 | 12 | 13 |
| 40-44 | 3 | 3 | 4 | 1 | 1 |
| 45-49 | 2 | 3 | 3 | 2 | 1 |
| 50-54 | 1 | 1 | 0 | 1 | 0 |
| 55-59 | 1 | 1 | 1 | 2 | 0 |
| >60 | 2 | 2 | 2 | 3 | 2 |

**Supplementary Table 7.** Size of repeats in the chloroplast genome of *C. subtilis* and its related species
