# Supplementary material for: Comparative and Phylogenetic Analysis Based on the Chloroplast Genome of Coleanthus subtilis (Tratt.) Seidel, a Protected Rare Species of Monotypic Genus
Source: Front Plant Sci. 2022 Feb 24;13:828467. doi: 10.3389/fpls.2022.828467 (PMC8908325; doi:10.3389/fpls.2022.828467)
Supplement: Supplementary file 1 [file Data_Sheet_1.zip › Supplementary Table/Supplementary Table 8.docx]

| **Region of repeats** | **Species** | | | | |
| --- | --- | --- | --- | --- | --- |
|  | *Phippsia algida* | *Coleanthus subtilis* | *Puccinellia nuttalliana* | *Sclerochloa dura* | *Zingeria biebersteiniana* |
| LSC | 25 | 25 | 28 | 28 | 26 |
| SSC | 3 | 3 | 2 | 3 | 0 |
| IR | 7 | 7 | 3 | 7 | 10 |
| LSC/IR | 6 | 6 | 8 | 8 | 8 |

**Supplementary Table 8.** Distribution of repeats in regions of the plastid genome.
