# Supplementary material for: Comparative and Phylogenetic Analysis Based on the Chloroplast Genome of Coleanthus subtilis (Tratt.) Seidel, a Protected Rare Species of Monotypic Genus
Source: Front Plant Sci. 2022 Feb 24;13:828467. doi: 10.3389/fpls.2022.828467 (PMC8908325; doi:10.3389/fpls.2022.828467)
Supplement: Supplementary file 1 [file Data_Sheet_1.zip › Supplementary Table/Supplementary Table 9.docx]

| **Repeats distribution in functional regions** | **Species** | | | | |
| --- | --- | --- | --- | --- | --- |
|  | *Phippsia algida* | *Coleanthus subtilis* | *Puccinellia nuttalliana* | *Sclerochloa dura* | *Zingeria biebersteiniana* |
| CDS | 16 | 16 | 19 | 18 | 17 |
| IGS | 10 | 11 | 6 | 12 | 11 |
| Intron | 4 | 4 | 4 | 4 | 4 |
| CDS/IGS | 3 | 2 | 3 | 4 | 3 |
| IGS/tRNA | 6 | 6 | 6 | 5 | 6 |
| IGS/intron | 2 | 2 | 3 | 3 | 3 |

**Supplementary Table 9.** Distribution of repeats in functional regions of the plastid genome.
